# The Role of Pain Catastrophizing and Pain Acceptance in Performance-Based and Self-Reported Physical Functioning in Individuals with Fibromyalgia and Obesity

**DOI:** 10.3390/jpm11080810

**Published:** 2021-08-19

**Authors:** Giorgia Varallo, Federica Scarpina, Emanuele Maria Giusti, Carlos Suso-Ribera, Roberto Cattivelli, Anna Guerrini Usubini, Paolo Capodaglio, Gianluca Castelnuovo

**Affiliations:** 1 Psychology Research Laboratory, Istituto Auxologico Italiano IRCCS, San Giuseppe Hospital, 28824 Verbania, Italy; g.varallo@auxologico.it (G.V.); r.cattivelli@auxologico.it (R.C.); anna.guerriniusubini@unicatt.it (A.G.U.); gianluca.castelnuovo@auxologico.it (G.C.); 2 Department of Psychology, Catholic University of Milan, 20123 Milan, Italy; 3 “Rita Levi Montalcini” Department of Neurosciences, University of Turin, 10124 Turin, Italy; f.scarpina@auxologico.it; 4 Unit of Neurology and Neurorehabilitation, Istituto Auxologico Italiano IRCCS, San Giuseppe Hospital, 28824 Verbania, Italy; 5 Department of Basic and Clinical Psychology and Psychobiology, Jaume I University, 12071 Castellon de la Plana, Spain; susor@uji.es; 6 Orthopaedic Rehabilitation Unit and Research Laboratory in Biomechanics and Rehabilitation, Istituto Auxologico Italiano IRCCS, San Giuseppe Hospital, 28824 Verbania, Italy; p.capodaglio@auxologico.it; 7 Department of Surgical Sciences, Physical and Rehabilitation Medicine, University of Turin, 10121 Turin, Italy

**Keywords:** fibromyalgia, physical functioning, obesity, pain catastrophizing, pain acceptance, chronic pain, rehabilitation, clinical psychology, performance-based test

## Abstract

Impaired physical functioning is one of the most critical consequences associated with fibromyalgia, especially when there is comorbid obesity. Psychological factors are known to contribute to perceived (i.e., subjective) physical functioning. However, physical function is a multidimensional concept encompassing both subjective and objective functioning. The contribution of psychological factors to performance-based (i.e., objective) functioning is unclear. This study aims to investigate the contribution of pain catastrophizing and pain acceptance to both self-reported and performance-based physical functioning. In this cross-sectional study, 160 participants completed self-report measures of pain catastrophizing, pain acceptance, and pain severity. A self-report measure and a performance-based test were used to assess physical functioning. Higher pain catastrophizing and lower pain acceptance were associated with poorer physical functioning at both self-reported and performance-based levels. Our results are consistent with previous evidence on the association between pain catastrophizing and pain acceptance with self-reported physical functioning. This study contributes to the current literature by providing novel insights into the role of psychological factors in performance-based physical functioning. Multidisciplinary interventions that address pain catastrophizing and pain acceptance are recommended and might be effective to improve both perceived and performance-based functioning in women with FM and obesity.

## 1. Introduction

Fibromyalgia (FM) is a complex chronic pain condition that predominantly affects women [1,2]. It is characterized by widespread pain, fatigue, cognitive impairment, and a decline in physical functioning. Although the aetiology of FM is still unclear, central sensitization might play a crucial role [3,4]. However, an interplay between genetic/biological and psychosocial factors appears to be necessary to explain the development and maintenance of this disease [5,6].

Impaired physical functioning is one of the most critical consequences associated with FM [1,7,8]. Reduced physical functional capacity is a significant obstacle to daily activities [9,10,11] and has a negative effect on the individual’s quality of life [3,12]. For example, women with FM have difficulties performing activities crucial to remaining physically independent, which increases their need for assistance from others [9]. 

The physical decline associated with FM is further exacerbated when individuals have comorbid obesity [13,14,15]. Obesity is an important condition to consider in FM owing to its high prevalence in this population, which can be partly attributed to the decreased activity levels in individuals with FM [7,13,16]. Obesity contributes to the disability burden associated with FM [13]. In particular, obesity is associated with increased pain severity and decreased physical functioning in FM [13,14,16,17,18]. Individuals with FM and obesity experience severe functional limitations owing to the combination of two issues: persistent pain related to FM and restricted movement imposed by obesity [13,19,20,21]. Thus, both conditions can interact with and exacerbate each other. Specifically, the chronic pain and fatigue associated with FM can lead to sedentary behavior, physical inactivity, and weight gain, which can negatively impact physical health and pain status, creating a vicious cycle [22].

In recent years, there has been a growing consensus on the importance of improving physical functioning rather than just reducing pain severity in people with chronic pain [23,24,25]. Consistent with this idea, efforts are being made to shift the focus of pain management from reducing pain to improving the quality of life [25,26,27]. As a consequence, modifiable psychological factors like pain-related cognitions and beliefs about experience of pain that partly explain individual differences in the adaptation to chronic pain, such as pain catastrophizing and pain acceptance, have gained ground in pain research in the past decades [28,29,30,31,32].

Pain catastrophizing is defined as a set of exaggerated and negative cognitive-emotional responses to actual or anticipated painful sensations [33]. According to the fear-avoidance model, which is a theoretical model developed to outline how pain-related cognitions influence pain experience [34,35,36], pain catastrophizing is a key cognitive factor associated with poor functioning in individuals with chronic pain. Indeed, the tendency to exaggerate and ruminate on pain experiences, combined with the tendency to feel helpless during pain episodes (i.e., pain catastrophizing), are known to lead to fear of movement and activity aversion [36]. As a result, individuals with chronic pain who tend to catastrophize engage in a variety of safety behaviors (i.e., movement avoidance, guarded movement) to prevent the worsening of pain symptoms [37]. Eventually, these behaviors lead to a cycle of avoidance; deconditioning; and, as a result, increased disability [7,38,39].

Pain catastrophizing has been observed in individuals with FM [40] and has been robustly associated with physical impairment in FM and other pain conditions [41,42,43,44]. However, the role of pain catastrophizing in individuals with chronic pain conditions and comorbid obesity remains less clear. Somers et al. reported higher levels of pain catastrophizing in individuals with severe obesity and osteoarthritis compared with individuals with less severe degrees of obesity and overweight [45]. Shelby and colleagues observed that higher levels of pain catastrophizing were associated with higher levels of disability in older adults with overweight, obesity, and osteoarthritis [46]. On the contrary, we recently observed no significant association between pain catastrophizing and physical disability in a sample of individuals with obesity and chronic low back pain [32]. These contradictory and heterogeneous findings could be attributed to the characteristics of participants in terms of age, clinical condition, and level of obesity, among other factors. However, the scarcity of evidence about the role of pain catastrophizing in individuals with FM and comorbid obesity justifies the need for more research.

Pain acceptance is another psychological factor that has received increasing attention in chronic pain research [47]. It refers to the willingness to experience painful sensations without attempting to avoid them, as well as the willingness to continue significant activities despite the presence of pain [48]. While the fear-avoidance model considers pain catastrophizing as a major contributor to pain-related disability, the psychological flexibility model of pain identifies pain acceptance as a key factor underlying individual differences in the pain adaptation [49]. The psychological flexibility model emphasizes the importance of an individual’s ability to flexibly change or persist with a behavior considering personal goals and values in a setting of competing psychological influences (such as acceptance of pain) and situational conditions [49]. Persisting in value-oriented behaviors despite uncomfortable experiences, such as pain, is essential to living a full and meaningful life according to this framework [50]. Indeed, pain acceptance appears to facilitate participation in valued activities and the pursuit of personal goals [51], which results in reduced pain interference with daily activities and less perceived disability [47,51,52].

The combination of both pain catastrophizing and pain acceptance might be important to consider in pain research. Previous studies have generally focused on the identification of psychological factors that have a negative impact on physical functioning in individuals with chronic pain [31,40,53,54,55,56]. However, in recent years, resilience factors have also gained ground in pain research [57]. Pain acceptance has emerged as a crucial component in the adaptation to chronic pain. Pain acceptance is, in fact, increasingly targeted in rehabilitative interventions that emphasize the promotion of resources rather than the restoration or modification of only negative and dysfunctional behaviors [58]. In summary, while pain catastrophizing may act as a barrier to a favorable adaptation to chronic pain (i.e., risk or vulnerability factor), pain acceptance might be a facilitator (i.e., resilience factor) [59]. 

To date, research into physical functioning in individuals with chronic pain and FM has mostly relied on self-report measures [25,41,60,61,62,63,64,65,66,67,68,69]. Performance-based measures, which focus on actual rather than perceived functional ability, have generally been neglected. Importantly, however, self-report and performance-based measures provide different, but complementary information about physical functioning [70]. Physical function is a multidimensional construct that should encompass both subjective (i.e., self-reported functional capacity) and objective (i.e., impaired movement) aspects [25,71,72,73]. While self-report measures assess what individuals “believe they can do’’, performance-based tests evaluate what they ‘’can actually do’’ [70]. The contribution of psychological factors tends to be greater for subjective aspects that involve a cognitive evaluation (such as perceived functioning) rather than actual performance in daily activities [74]. Therefore, past research might have revealed strong associations between psychological factors and self-report measures of physical functioning because they assessed subjective perceptions. Importantly, performance-based and self-reported physical functions are not significantly correlated in individuals with heterogeneous musculoskeletal chronic pain [71]. In addition, a study highlighted that individuals with FM report worse subjective physical functioning compared with objective performance [75]. Therefore, research that evaluates and compares the contribution of psychological factors to perceived and performance-based functioning outcomes is necessary to advance interdisciplinary interventions for chronic pain.

In summary, the goal of the present study is to investigate the contribution of pain catastrophizing (vulnerability factor) and pain acceptance (resilience factor) to the physical functioning of individuals with FM and comorbid obesity. We hypothesized that pain catastrophizing [41,42,43,52,56] would be associated with reduced physical functioning, while pain acceptance would be associated with better physical functioning [47,60,62,76]. We also hypothesized that the contribution of psychological variables will be greater for perceived versus performance-based functioning [74].

## 2. Materials and Methods

### 2.1. Procedure and Participants

A cross-sectional study was conducted. An a priori power analysis was performed using G. Power (version 3.1.9.4) [77] to calculate the required sample size to detect statistical significance. Setting a conservative small-to-medium effect size of the predictors (0.10), an alpha of 0.05, and a power of 0.90, a minimum sample size of *n* = 130 was required to detect the hypothesized effects. 

From January 2019 to September 2019, 160 women were consecutively recruited at the start of a one-month-long hospitalization program for weight loss and physical therapy at the Orthopaedic Rehabilitation Unit of the IRCCS Istituto Auxologico Italiano (Piancavallo, Italy). Data collection was conducted during the first week of the diagnostic assessment, before starting a physical therapy and nutritional rehabilitative program for weight loss. 

Participants were included in this study if they (a) had a FM diagnosis provided by a rheumatologist according to Wolfe et al. criteria [1]; (b) met the American College of Rheumatology (ACR) Research Criteria for fibromyalgia [78,79], as measured by the Fibromyalgia Survey Questionnaire in its Italian version [53]; (c) were over 18 years; and (d) were able to sign an informed consent form and could complete the questionnaires. 

Patients were excluded if they (a) had psychiatric disorders with psychotic symptoms or severe personality disorders; (b) had previously or were currently receiving psychological treatment for FM; and (c) had comorbid acute pain conditions or comorbid chronic pain conditions different from FM.

### 2.2. Measures

Sociodemographic and clinical data. Participants completed a self-report protocol that included age, weight (in kilograms), and height (in centimeters), job status (i.e., employed, unemployed, or disability pension), current opioid use, and pain duration (in years).

Fibromyalgia symptomatology. The Italian version of the fibromyalgia survey questionnaire (FSQ) [53] was used to assess the ACR fibromyalgia research criteria [78,79]. This measure, which is recommended in [78,80], was administered to evaluate the level of symptomatology required to confirm the diagnosis of FM. Individuals must meet the following research criteria: (1) widespread pain index ≥7 and symptom severity scale score ≥5 OR widespread pain index of 4–6 and symptom severity scale score ≥9; (2) symptoms have generally been present for at least 3 months.

Pain severity. The numeric pain rating scale (NPRS) [81] was used to assess pain severity levels. It consists of an 11-point scale (anchors 0 = “no pain”; 10 = “worst possible pain”). The Numeric Pain Rating Scale is a well-validated and widely used measure of pain severity in chronic pain conditions [82].

Pain catastrophizing. The pain catastrophizing scale (PCS) [83] is a self-report measure of pain-related catastrophic thinking. It consists of 13 items on a five-point Likert scale (from 0 = “not at all” to 4 = “all the time”). The total score ranges from 0 to 52, with higher scores indicating higher levels of pain catastrophizing [84]. The Italian version [84] has psychometric properties comparable with the original version. In the current study, internal consistency was excellent (Cronbach’s α = 0.89).

Pain acceptance. The chronic pain acceptance questionnaire (CPAQ) is a measure of pain acceptance [85,86,87] that consists of 20 items scored on a 7-point Likert scale (0 = “never true” to 6 = “always true”). The maximum total score is 120, with higher scores indicating greater acceptance. The Italian version of the questionnaire was used [88], which, in line with the original version, has obtained good psychometric properties. In the present study, the internal consistency of this measure was excellent (Cronbach’s α = 0.90).

Self-report physical functioning limitations. Self-reported physical functioning limitations were assessed using the physical functioning subscale of the fibromyalgia impact questionnaire—revised (PF-FIQR). The FIQR is a 21-item measure. The items use an 11-point numeric rating scale ranging from 0 to 10, with 10 representing “worst” functioning scores. All questions are related to the functioning of the last 7 days. Higher total scores indicate a higher impact of the disease and worse functioning. The FIQR assesses three domains: (a) ‘physical function’, (b) ‘overall impact’, and (c) ‘symptoms’. The total score, which assesses the impact of FM on overall functioning, can also be computed. Considering the aim of the present study, the physical function subscale was used. Specifically, the physical functioning subscale of the FIQR includes nine items developed to evaluate the level of physical impairment. This scale has been used in previous research [38,75,89,90]. Scores on the physical functioning subscale of the FIQR range from 0 to 30, with higher scores indicating worse physical functioning. The FIQR and its subscales have good psychometric properties and good discriminant ability [38]. In the current study, internal consistency was good (Cronbach’s α = 0.82).

Performance-based physical functioning. The 6-min walking test (6MWT) is a performance-based test used to evaluate the ability to walk and is a simple and inexpensive measure of physical functioning. The 6MWT has been used in research with persons with FM [38,90,91] with good applicability and reliability findings [38,92]. In this test, the participant is required to walk for six minutes along a rectangular course of 45.7 m. Walking distance in meters is measured with higher scores, indicating better physical performance. The distance walked during the 6MWT has been found to be shorter in females with FM compared with healthy women (i.e., discriminant validity) [9]. Furthermore, the 6MWT has good reproducibility and is recommended for the assessment of walking ability in individuals with obesity [93]. Women with obesity have also been found to walk significantly shorter distances during the 6MWT compared with their normal-weight counterparts [94,95].

### 2.3. Statistical Analysis

Counts and percentages were used to describe categorical variables, whereas means and standard deviations were used to describe continuous variables. Height in meters and weight in kilograms were used to compute body mass index (i.e., BMI, as the weight in kilograms divided by the square of height in meters (kg/m^2^) [96]), which is considered as an indicator of obesity (BMI ≥ 30) [96].

Pearson bivariate analyses were performed to evaluate the associations between the numeric pain rating scale (i.e., pain severity), the pain catastrophizing scale (i.e., pain catastrophizing), the chronic pain acceptance questionnaire (i.e., pain acceptance), the physical functioning scale of the fibromyalgia impact questionnaire (i.e., self-reported physical functioning), and the 6-min walking test (i.e., performance-based physical functioning).

To answer our research question, we used multivariate hierarchical regression analyses, as done in previous studies [28,29,33,34]. Before the multivariate regression, we checked the assumptions of normality, linearity, homoscedasticity, and multicollinearity of the data. To evaluate the contribution of pain catastrophizing and pain acceptance, self-reported physical functioning limitations and performance-based physical functioning were independently introduced as dependent factors in two separate regressions. In both models, age [97,98], opioid use [98,99], and pain duration [100,101] were entered into the first step as covariates to control for their relationship with the outcome measures. Pain severity was entered in the second step, because of its association with physical functioning [38,102]. Pain catastrophizing and pain acceptance were entered simultaneously in the third and last step. The change in explained variance (∆R^2^) was used to evaluate the additional variance of the dependent variables accounted for by the variables included in each block. The significance was evaluated using a criterion of *p* < 0.05. Data were analyzed using the Jamovi software [103].

## 3. Results

The participant’s characteristics are reported in Table 1.

### 3.1. Correlations

Pearson’s correlations between measures are reported in Table 2. Pain catastrophizing and pain acceptance were significantly and negatively correlated (*r* = −0.49, *p* < 0.001). Pain catastrophizing was significantly and positively associated with pain severity (*r* = 0.42, *p* < 0.001), perceived limitations in physical functioning (*r* = 0.43, *p* < 0.001), and performance-based physical functioning (*r* = −0.57, *p* < 0.001). On the contrary, pain acceptance was significantly and negatively related to pain severity (*r* = −0.39, *p* < 0.001) and limitations in physical functioning (*r* = −0.47, *p* < 0.001), and linked to improved performance-based physical functioning (*r* = 0.52, *p* < 0.001). Perceived physical limitations and performance-based physical functioning were correlated in the negative direction (*r* = −0.53, *p* < 0.001), because higher scores on the self-report physical functioning limitations measure correspond to greater disability, while higher scores on the performance-based test reflect better physical functioning.

### 3.2. Hierarchical Regression Relative to Self-Reported Physical Functioning Limitations

The first multivariate hierarchical regression was conducted using self-report physical functioning limitations as the dependent variable (Table 3). The demographic characteristics (i.e., pain duration, age, current opioid use, and body mass index) included in the first step explained a nonsignificant 4% (R^2^ = 0.04) variance in self-report physical functioning limitations in the first step (F (4155) = 1.78; *p* = 0.14). Pain severity, which was included in the second step, significantly contributed an additional 12% of the explained variance (ΔF (1154) = 22.60; *p* < 0.001; ΔR^2^ = 0.12) of self-report physical functioning limitations. The third step, which included pain catastrophizing and pain acceptance, significantly explained an additional 17% of the variance of self-reported physical functioning limitations (ΔF (2152) = 20.10; *p* < 0.001; ΔR^2^ = 0.17).

In the final model, opioid use (B = 2.66, *p* = 0.006), pain acceptance (B = −0.13, *p* < 0.001), and pain catastrophizing (B = 0.12, *p* ≤ 0.001) contributed unique variance to the prediction of self-reported physical functioning limitations. Pain severity, which was significantly associated with self-report physical functioning limitations in the second step, became nonsignificant when accounting for the contribution of pain catastrophizing and pain acceptance.

### 3.3. Hierarchical Regression Relative to Performance-Based Physical Functioning

A second multivariate hierarchical regression was conducted using performance-based physical functioning as the dependent variable (Table 4). The first step, which included demographic variables (i.e., pain duration, age, current opioid use, body mass index), explained a significant 12% (R^2^ = 0.12) variance in performance-based physical functioning (F (4155) = 5.23, *p* ≤ 0.001). In the second step, pain severity contributed an additional 14% variance in performance-based physical functioning (ΔF (1154) = 28.9; *p* < 0.001; ΔR^2^ = 0.139). Next, pain catastrophizing and pain acceptance included in the third step significantly explained an additional 23% variance of performance-based physical functioning (ΔF (2152) = 33.3; *p* < 0.001; ΔR^2^ = 0.226).

Pain duration, current opioid use, and pain severity were significantly associated with performance-based physical functioning in the final model. Additionally, both pain catastrophizing (B = −0.364; *p* < 0.001) and pain acceptance (B = 0.272; *p* ≤ 0.001) uniquely and significantly contributed to performance-based physical functioning.

## 4. Discussion

In this study, we aimed to explore the association between pain catastrophizing and pain acceptance with self-reported and performance-based physical functioning in individuals with FM and obesity. We confirmed our hypothesis that higher pain catastrophizing and lower pain acceptance would be significantly associated with poorer physical functioning when assessed with a self-reported and a performance-based measure. Specifically, both pain catastrophizing and pain acceptance were significant predictors of self-reported and performance-based physical functioning, even after controlling for body mass index, pain duration, current opioid use, and pain severity. Contrary to our hypotheses, however, the contribution of psychological variables was stronger for performance-based compared with self-reported physical functioning.

Research has repeatedly shown that pain catastrophizing and pain acceptance are important predictors of self-reported physical functioning in samples with acute [104,105] and chronic pain [105,106], including FM [41,107,108,109,110]. Our findings are consistent with the previous evidence. Previous research, however, has primarily focused on subjective measures of functional capacity. Our work contributes to the literature by combining a self-reported measure with a performance-based test that provides a more objective evaluation of physical functioning. The present study revealed that psychological factors contribute not only to perceived physical functioning, but also to actual functioning. Importantly, both pain catastrophizing and pain acceptance uniquely contributed to the prediction of self-reported and performance-based functioning, implying that the two factors are likely to influence physical functioning via distinct pathways. Ultimately, the fact that the two psychological factors contributed significantly to physical functioning when included in the same multivariate analysis supports the idea that they should both be independently targeted in multidisciplinary interventions. In addition, this contributes to the validity of different psychological models to understand the pain experience, such as the fear-avoidance model and the psychological flexibility model.

Individuals who catastrophize tend to appraise pain as a catastrophic and harmful experience and frequently ruminate and feel hopeless about it [43,111]. Based on the present results, the tendency to catastrophize about pain might negatively affect both the individual’s perception of what they are capable of accomplishing in terms of physical performance (i.e., what I think I can do) as well as their actual physical performance (i.e., what I can do). We propose a mechanism to explain why this might occur. Pain catastrophizing might increase the level of attention and awareness of painful sensations [112], thus increasing protective behaviors. Individuals with chronic pain who catastrophize engage in a variety of safety behaviors (e.g., activity avoidance, movement restriction, and guarded movement) to prevent the worsening of pain symptoms [37]. Obesity, in turn, might increase avoidance in chronic musculoskeletal pain conditions [113]. In particular, the belief that excessive weight causes additional damage or increases pain might play an additional role in restricting activity, which, combined with skin friction, discomfort, and respiratory difficulties [114], might result in the avoidance of movements and, in turn, impediment to weight loss [113].

On the other hand, the willingness to persist with important activities without attempting to avoid pain (i.e., acceptance) might have a positive influence on both subjective and actual physical functioning [47,51,52]. Individuals who accept pain as an unpleasant experience that they are willing to undergo in order to achieve their goals might be more likely to move and engage in valued activities despite the pain. In addition, acceptance might facilitate the adaptation to chronic pain by focusing on one’s personal goals rather than on pain control, thereby preventing the implementation of pain-avoidance behaviors [47,51,52]. Taking all the previous points into account, reducing pain catastrophizing and enhancing pain acceptance by implementing psychological interventions might help individuals with chronic pain to reduce the adoption of unnecessary and detrimental protective behaviors that perpetuate a cycle of avoidance, deconditioning, and increased disability [7,38,39].

Interestingly, we observed that the self-reported measure of physical function and the performance-based test were significantly and moderately related. Our results are consistent with those of Mannerkorpi and colleagues [38], but not with the findings of Greenberg and colleagues, who found no significant relationship between subjective and objective measures of physical functioning in individuals with heterogeneous chronic pain [71]. These discrepancies could be owing to differences in sample characteristics or measurements used across studies, or they might suggest that the relationship between subjective and objective components of physical functioning might be modulated differently in clinical conditions. While acknowledging this, owing to the scarcity of existing studies, more research is still needed to determine the extent to which self-report and objective measures of functioning are associated in different populations. Such studies are important because they investigate whether a subjective assessment of physical functioning can be used as a reliable alternative measure in objective tests of physical functioning, which are generally more time-consuming.

An interesting and unexpected finding was that the contribution of psychological factors was greater for performance-based physical functioning as opposed to self-reported physical functioning, while psychological aspects have a greater impact on factors that require cognitive evaluation [74]. One possible explanation for the findings is that the performance-based test might be interpreted as both pain-provoking and/or actually painful to perform. Thus, attention directed at a potential or actual pain-inducing movement might activate the repertoire of pain-related cognitive/coping strategies in a more prominent and influential way to motivate different behaviors. For example, individuals who tend to catastrophize about pain might be encouraged to restrict movement when they experience pain in real-world contexts, while those who accept pain might be prompted to persist in movement in this same scenario. More studies are needed to confirm this hypothesis, which could lead to an interesting line of future research.

Body mass index was not significantly related to self-reported and performance-based physical functioning. This result contradicted previous evidence [13,115,116]. It should be noted, however, that the variability in body mass index was limited in our sample because only individuals with obesity were included. In addition, recent studies have argued that body mass index may be an inadequate and oversimplified measure that does not properly capture the complexity of obesity [117,118]. Different indices of obesity could be used in future studies, such as the level of adiposity and the distribution of adipose tissue [119,120].

Among other control factors, current opioid use was found to be significantly associated with both self-reported and performance-based physical functioning. Individuals who are currently on opioid therapy might rely on this medication because of their low level of both subjective and objective physical functioning. Instead, pain duration and pain severity were only significantly related to performance-based physical functioning. The biological function of pain is to alter behavior by prioritizing protection and avoidance [121], and it seems likely that pain has a more pronounced effect on physical performance than on its perception [122]. Relative to pain duration, it is possible that individuals with a longer history of chronic pain have implemented dysfunctional coping strategies over the years (such as avoidance of movement and activities due to pain persistence despite treatment) that lead to the development of deconditioning and disuse, thus worsening the actual ability to move [35,123].

The findings of this study could have several clinical implications. According to our results, measures of pain catastrophizing and pain acceptance should be included in the assessment of biopsychosocial aspects of pain in FM and obesity to provide a more complete picture of the factors that significantly affect physical functioning. Furthermore, our findings suggest that pain catastrophizing and pain acceptance should be treatment targets in psychological evidence-based intervention to improve physical functioning in individuals with FM, and obesity should include pain catastrophizing and pain acceptance as treatment targets. In support of this hypothesis, Baranoff and colleagues [47] highlighted how changes in pain catastrophizing and pain acceptance accounted for changes in self-reported and performance-based disability in individuals with chronic pain, primarily located in the lower back. Importantly, because both pain catastrophizing and pain acceptance are significant predictors of both perceived and actual physical functioning, multidisciplinary interventions targeting both factors are likely to improve both aspects of functional capacity. More research is required to test this hypothesis in individuals with FM, especially when it is associated with obesity.

Finally, it is important to note that the willingness to move and engage in physical activity is critical in the management of both FM and obesity. Physical activity improves physical functioning in individuals with FM [124,125,126,127,128,129] as well as in those with obesity [130,131,132]. Consequently, current and previous research supports the idea that it might be beneficial to reduce pain catastrophizing and enhance pain acceptance in order to promote adherence to physical activity and a healthy, active lifestyle in individuals with FM and obesity. More research is needed to determine what other factors might be important in promoting physical activity compliance.

This study has some limitations. We did not include a control group (for example, individuals who are only affected by FM or obesity). In addition, we focused only on two psychological factors. While both are important factors according to the pain literature, other psychological factors, such as kinesiophobia [31] or pain self-efficacy [133], might also play a role in physical functioning. Furthermore, while the self-report questionnaire used referred to a period in the past (e.g., the previous week), the performance-based measure was based on the present moment. While most available measures refer to this timeframe, the development of self-reported measures that refer to the current time of assessment could be used to mitigate this mismatch. In addition, the pain severity measure used in this study assesses the intensity of perceived pain at the time of completion. As there can be variability in the levels of perceived pain in fibromyalgia even within a single day [134,135,136], measures that assess medial pain intensity over a week could be implemented to address this limitation. Finally, because we focused on individuals with FM and obesity as a comorbid condition, the findings might not be generalizable to other populations.

## 5. Conclusions

Our findings suggest that pain catastrophizing and pain acceptance should be addressed to improve performance-based and self-reported physical functioning in individuals with FM and obesity. If these components are ignored, rehabilitative interventions may neglect critical factors associated with the maintenance of poor physical functioning and physical health.

## Figures and Tables

**Table 1:** Means and standard deviations of sociodemographic characteristics and clinical measures. The theoretical range and actual range are reported. *n* = 160.

Sociodemographic Characteristics			*n* = 160
Age in years (mean ± SD)			43.6 ± 7.25
Body mass index (mean ± SD)			44.3 ± 7.15
Pain duration in years (mean ± SD)			7.08 ± 2.70
Current opioid use (%)			13.1%
Employed (%)			71.9%
Full-time			22.5%
Part-time			49.4%
Clinical measures	Theoretical range	Sample’s range	Mean ± SD
Widespread pain index	0–19	7–18	13.8 ± 2.70
Symptoms’ severity	0–12	5–11	8.13 ± 1.85
Numeric pain rating scale	0–10	3–9	5.67 ± 1.58
Pain catastrophizing scale	0–52	0–44	27.3 ± 10.3
Chronic pain acceptance questionnaire	0–120	21–74	51.7 ± 11.2
Physical functioning subscale	0–30	9–29	17.7 ± 4.77
6-Min walking test in meters	NA *	201–402	306 ± 59.4

Note. * NA: Not applicable.

**Table 2:** Pearson correlation coefficients between study variables. *n* = 160.

	1	2	3	4
1. Pain severity (NPRS)	-			
2. Pain catastrophizing (PCS)	0.42 *	-		
3. Pain acceptance (CPAQ)	−0.39 *	−0.49 *	-	
4. Self-reported physical functioning limitations (PF-FIQR)	0.36 *	0.43 *	−0.47 *	-
5. Performance-based physical functioning (6MWT)	−0.38 *	−0.57 *	0.52 *	−0.53 *

Note. NPRS: numeric pain rating scale; PCS: pain catastrophizing scale; CPAQ: chronic pain acceptance questionnaire; PF-FIQR: physical functioning subscale of the firbomyalgia impact questionnaire; 6MWT: 6-minute walking test. * *p* < 0.001.

**Table 3:** Multivariate hierarchical regression predicting self-report physical functioning limitations.

	Step 1		Step 2		Step 3	
Factors	B (SE)	*p*	B (SE)	*p*	B (SE)	*p*
Pain duration	−0.04 (0.14)	0.792	0.02 (0.13)	856	−0.07 (0.12)	0.566
Age	−0.01 (0.05)	0.826	0.02 (0.05)	0.707	−0.01(0.04)	0.929
Current opioid use	2.86 (1.13)	0.012	2.70 (1.06)	0.110	2.66 (0.95)	0.006
Body mass index	0.01 (0.05)	0.897	−0.04 (0.05)	0.400	−0.09 (0.23)	0.054
Pain severity			1.09 (0.23)	<0.001	0.44 (0.23)	0.060
Pain catastrophizing					0.12 (0.04)	0.003
Pain acceptance					−0.13 (0.03)	<0.001

**Table 4:** Multivariate hierarchical regression predicting performance-based physical functioning.

	Step 1		Step 2		Step 3	
Factors	B (SE)	*p*	B (SE)	*p*	B (SE)	*p*
Pain duration	−3.85 (1.66)	0.022	−4.65 (1.54)	0.003	−3.37 (1.30)	0.010
Age	−1.07 (0.62)	0.089	−1.16 (0.58)	0.045	−0.81 (0.49)	0.099
Current opioid use	−37.79 (13.47)	0.006	−35.68 (0.59)	0.005	−35.99 (10.43)	<0.001
Body mass index	−1.02 (0.63)	0.109	−0.36 (0.59)	0.544	0.29 (0.51)	0.574
Pain severity			−14.46 (2.69)	<0.001	−5.05 (2.54)	0.048
Pain catastrophizing					−2.21 (0.43)	<0.001
Pain acceptance					1.45 (0.37)	<0.001

## Data Availability

The data presented in this study are available on request from the corresponding author. The data are not publicly available because they contain information that could compromise the privacy of research participants.
